# A Rare Adverse Effect of Olanzapine: Striae Development Without Weight Gain

**DOI:** 10.1192/j.eurpsy.2026.11786

**Published:** 2026-07-31

**Authors:** M. Altın Kara, K. C. Can

**Affiliations:** 1 Psychiatry, İstanbul Tuzla State Hospital, İstanbul; 2 Psychiatry, Ankara University Faculty of Medicine, Ankara, Türkiye

## Abstract

**Introduction:**

Olanzapine is an atypical antipsychotic agent that acts as an antagonist of serotonin and dopamine receptors. Olanzapine has been used for a long time, and many of its common adverse effects have been thoroughly documented.

**Objectives:**

In this report, we present a case of olanzapine-induced striae without any associated increase in weight.

**Methods:**

A female patient in her 20s was referred to our clinic by the internal medicine department due to striae that appeared after starting olanzapine. One week after initiating olanzapine treatment, the patient developed purple-violet striae that first appeared in the upper leg area, and as the medication continued, the striae became more widespread on both legs and arms (Image 1-2). Consequently, the medication was discontinued. Following the discontinuation, it was observed that the color of the purple-violet striae gradually faded, and no new lesions appeared.

**Results:**

In an extensive literature review, only one article reporting olanzapine-induced striae was found. According to the report, all cases were adolescents and developed striae involving their hips, thighs, lower legs, breasts, and upper arms, which persisted after olanzapine was discontinued. Blomgren et al. report that four more cases were reported to the WHO’s International Drug Monitoring Program (Olanzapine-associated striae: 5 cases in adolescents in Sweden. Reactions Weekly 2001). However, in these nine cases, the dose and duration of olanzapine, as well as the possibility of weight gain, were not reported. In this paper, we present the first case of an adult patient who developed widespread striae with a daily dose of 5 mg/d olanzapine for two months without weight change, and new striae did not form after the drug was discontinued.

**Image 1:**
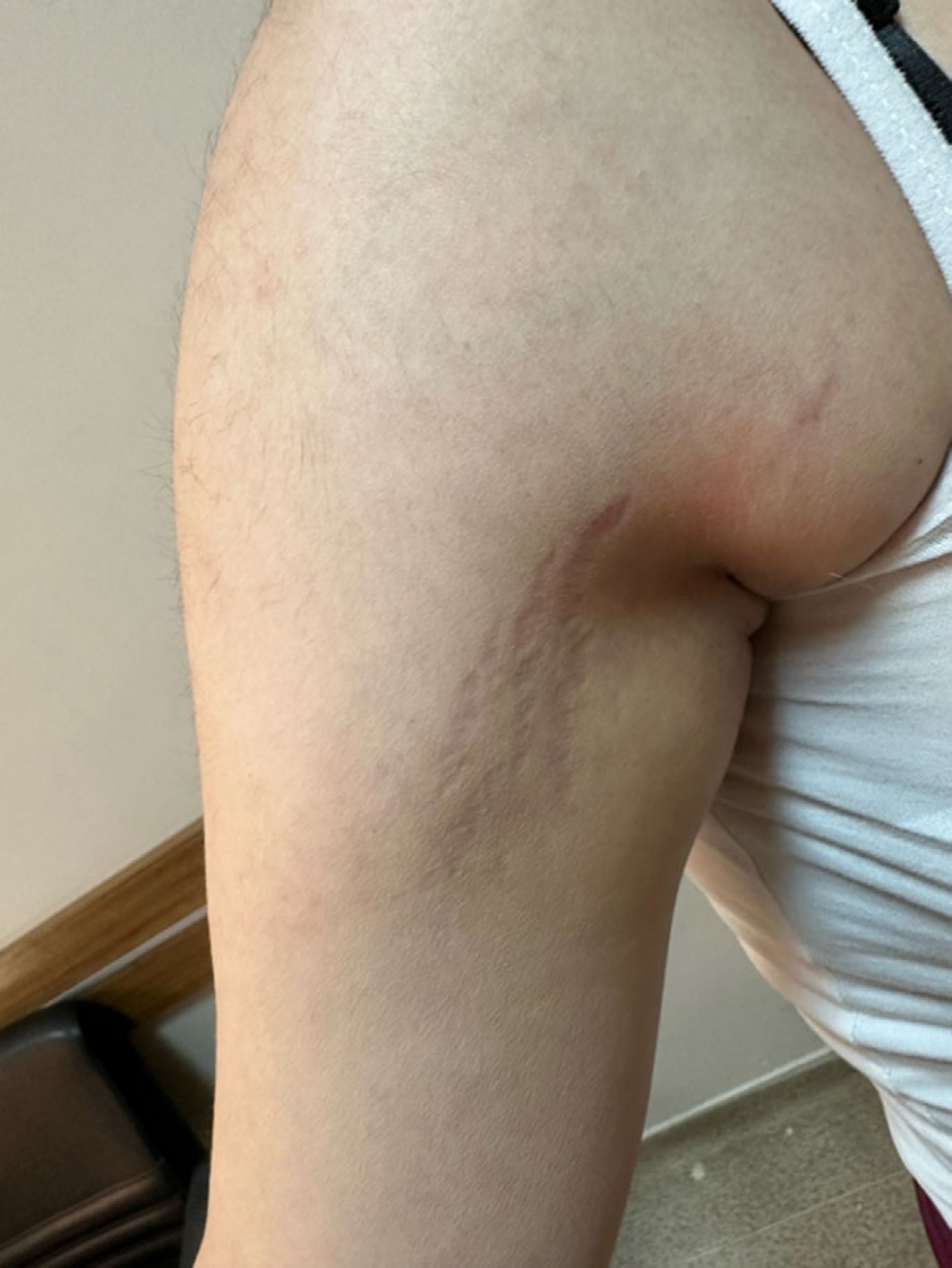

**Image 2:**
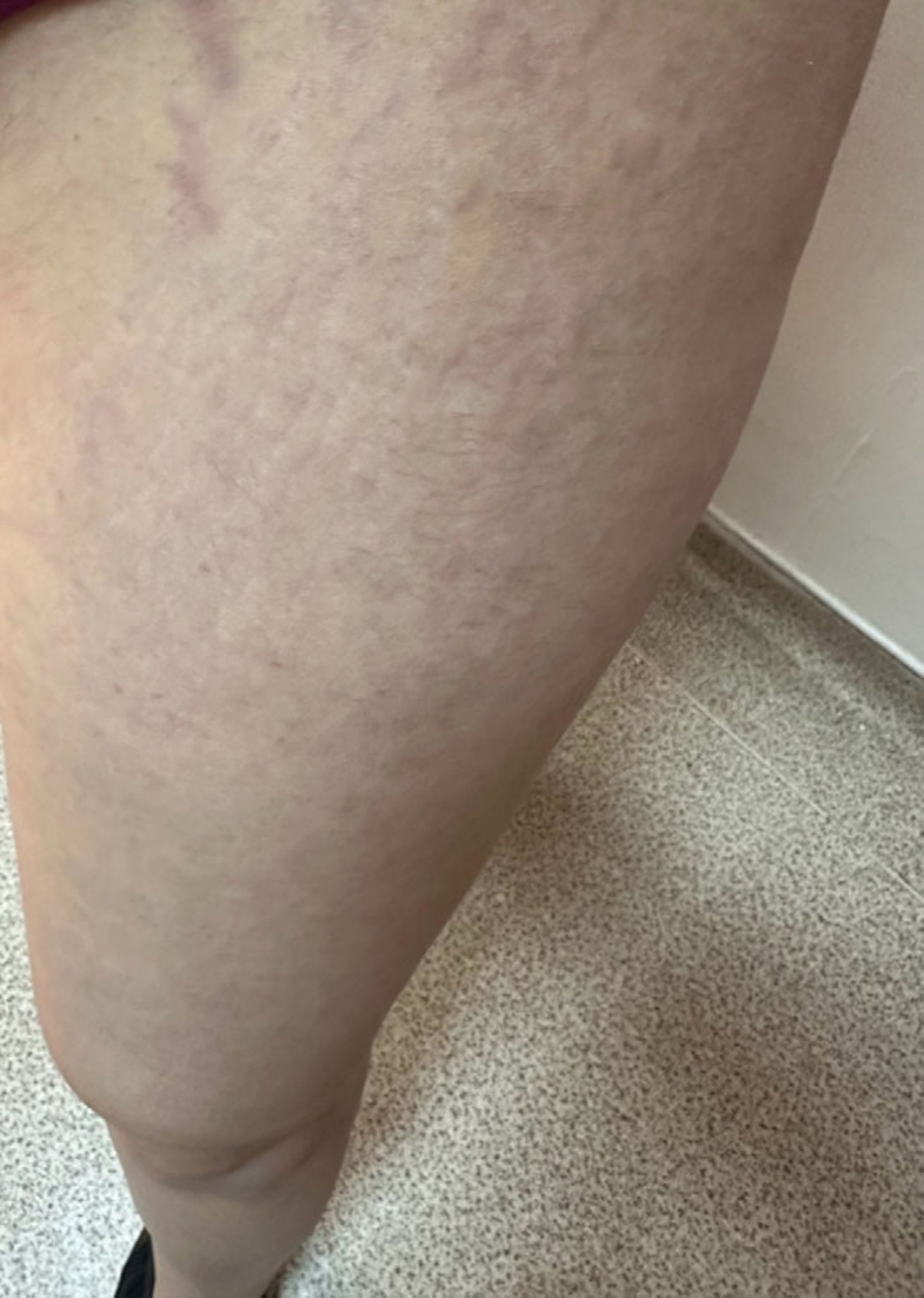

**Conclusions:**

This case adds to the literature by suggesting a possible association between olanzapine use and the development of striae in some patients. It is important for healthcare providers to be aware of this potential side effect when prescribing olanzapine. Early recognition and management of the rare adverse effects can help improve patient outcomes and prevent unnecessary distress.

**Disclosure of Interest:**

None Declared

